# Viral infections in prostate carcinomas in Chilean patients

**DOI:** 10.1186/s13027-015-0024-y

**Published:** 2015-09-01

**Authors:** Hector Rodríguez, Jorge Levican, Juan P. Muñoz, Diego Carrillo, Mónica L. Acevedo, Aldo Gaggero, Oscar León, Tarik Gheit, Omar Espinoza-Navarro, Jorge Castillo, Iván Gallegos, Massimo Tommasino, Francisco Aguayo

**Affiliations:** Anatomy and Development Biology Program, Institute of Biomedical Sciences (ICBM), Faculty of Medicine, Universidad de Chile, Santiago, Chile; Virology Program, Institute of Biomedical Sciences (ICBM), Faculty of Medicine, Universidad de Chile, Santiago, Chile; Infections and Cancer Biology Group, International Agency for Research on Cancer (IARC), Lyon, France; Biology Department, Universidad de Tarapacá, Tarapacá, Chile; Pathological Anatomy Department, Barros Luco-Trudeau Hospital, Universidad de Chile, Santiago, Chile; Pathological Anatomy Service, Clinical Hospital, Universidad de Chile, Santiago, Chile

**Keywords:** Prostate, Cancer, Virus

## Abstract

**Background:**

A few viruses have been detected in prostate cancer, however their role in the development of this malignancy has not been determined. The aim of this study was to analyze the presence and functionality of human papillomavirus (HPV) and polyomaviruses (BKPyV and JCPyV) in prostate carcinomas in Chilean patients.

**Methods:**

Sixty-nine primary prostate carcinomas were analyzed for the presence of HPV, BKPyV and JCPyV using standard polymerase chain reaction protocols. In addition, when samples were positive for HPyV, large T antigen (TAg) transcripts were analyzed using reverse transcriptase PCR.

**Results:**

HPV and JCPyV were not detected in any specimens (0/69, 0 %); whereas, BKPyV was detected in 6/69 PCas (8.7 %). We did not find a statistically significant association between the presence of BKPyV and age (*p* = 0.198) or Gleason score (*p* = 0.268). In addition, 2/6 (33 %) BKPyV positive specimens showed detectable levels of TAg transcripts.

**Conclusions:**

There was no association between HPV or JCPyV presence and prostate cancer development. The presence of BKPyV in a small subset of prostate carcinomas in Chilean patients could indicate that this virus plays a potential role in prostate cancer development and requires further investigation.

## Introduction

Prostate cancer (PCa) is the second most common cancer worldwide [1], and is particularly common in industrialized countries. In Chile, the incidence rate of PCa is 27.9/100,000 inhabitants and its mortality rate is 15.6/100,000 inhabitants [1]. Human papillomavirus (HPV) and polyomaviruses (HPyV) are small non-enveloped DNA viruses that have been detected at variable frequencies in PCas worldwide. However, the etiological role of these viruses has only been established in specific tumors. For example, high-risk HPVs are the causal agent of cervical, anogenital and a subset of oropharyngeal carcinomas [2]. In addition, there is convincing evidence that Merkel cell polyomavirus (MCPyV) is the etiological agent of Merkel cell carcinomas [3]. Although the DNA of both HPV and HPyV has been detected in PCas, there is high variability in the detection rates between different studies. Nevertheless, the mere detection of viral DNA fragments in clinical specimens is not a sufficient to indicate causality. Previous reports have described the presence of HPV in biopsies of PCa and prostatic benign hyperplasia [4–8]. However, a recent meta-analysis concluded that high-risk HPV-16 and −18 did not contribute to increased PCa risk [9]. The reported frequencies of HPyVs, and in particular BKPyV, in PCas have been inconsistent [10–13]; while other studies have proposed that BKPyV is not involved in PCa [14, 15]. JCPyV is the etiologic agent of progressive multifocal leukoencephalopathy (PML), a neurodegenerative disease [16]. It has been suggested that the presence of JCPyV correlates with various types of human neoplasm, including colorectal, gastric, prostatic, oesophageal, brain and bronchial carcinomas and B cell lymphomas [17]. However, an oncogenic role for this virus has not been definitively established. Taken together, the definitive role of viral infections, such as HPV, BKPyV and JCPyV, in prostate carcinogenesis has not been established. Thus the aim of this study was to determine the presence of HPV, BKPyV and JCPyV in PCas in patients at two public hospitals in Santiago, Chile, and to analyze the expression levels of viral oncogenes in virus-positive PCas samples.

## Materials and methods

### Clinical samples

For this retrospective study, formalin-fixed and paraffin-embedded specimens from PCas confirmed by histological analysis were used. Samples were collected at San Borja Arriarán Hospital (thirty one samples from 2004 to 2012) and at José Joaquin Aguirre Hospital (thirty eight samples, from 2005 to 2009) in Santiago, Chile. The participants did not provide their written or verbal informed consent to participate in this study. Under Chilean law, informed consent is not necessary if the analysis does not pertain to the human genome. Only viral DNA sequences were analyzed in this study, which was approved by the Ethics Committee of the José Joaquín Aguirre Hospital, Faculty of Medicine, Universidad de Chile.

### DNA extraction and detection of viral infections

Paraffin tissue sections were incubated with digestion buffer (10 mM Tris–HCl pH 7.4, 0.5 mg/mL proteinase K and 0.4 % Tween 20) at 37 °C for 3 h. Afterwards, the samples were incubated for 10 min at 95 °C, centrifuged for 2 min at 14,000 rpm and immediately placed on ice. After centrifugation, the aqueous phase was transferred to a new tube [18].

HPV typing was performed by a type-specific multiplex genotyping (TS-MPG) assay, which combines multiplex PCR and bead-based Luminex Technology, as previously described [19–21]. This assay is able to detect DNA from 19 alpha mucosal HPV types (16, 18, 26, 31, 33, 35, 39, 45, 51, 52, 53, 56, 58, 59, 66, 68, 70, 73 and 82). In addition, we used conventional PCR with the GP5+/6+ primers as an alternative technique for HPV detection. DNA quality was determined by PCR using the betaglobin gene fragment as a marker with the following primers: PCO3 5′-ACACAACTGTGTTCACTAGC-3′ and PCO4 5′-CAACTTCATCCACGTTCACC-3′. The amplification program was as follows: denaturation at 95 °C for 5 min, 45 cycles with a cycling profile of 95 °C for 30 s, 52 °C for 30 s, 72 °C for 30 s and final extension at 72 °C for 5 min. For HPV amplification we used the following primers: GP5+ 5′- TTTGTTACTGTGGTAGATATCAC-3′ and GP6+ 5′- GAAAAATAAACTGTAAATCATATTC-3′. The PCR conditions were as follows: denaturation at 95 °C for 5 min, 45 cycles with a cycling profile of 95 °C for 1 min, 52 °C for 2 min, 72 °C for 1.5 min and a final extension at 72 °C for 5 min. The amplification products were stained with MasterSafe (Promega), visualized under UV transillumination and photographed.

BKPyV detection was conducted using qPCR with an intercalating dye format as previously reported [22]. Briefly, a 127 bp fragment of the BKPyV genome was amplified with the following BK127-F and BK127-R primers: BK127-F: 5′-GCA GCT CCC AAA AAG CCA AA-3′ and BK127-R: 5′-CTG GGT TTA GGA AGC ATT CTA-3′. The 20 μL final volume reactions contained 1X LightCycler® FastStart DNA Master SYBR Green I (Roche Diagnostics, Indianapolis, IN, USA), 0.5 μM of each primer, 3.0 μM MgCl_2_ and 5 μL of DNA template. The PCR reaction was run for 40 cycles as follows: denaturation as 94 °C for 10 s, annealing at 62 °C for 10 s and extension at 72 °C for 10 s, with an initial denaturation at 95 °C for 15 min and final extension at 72 °C for 10 min. The amplification products were subjected to a melting profile and the specific product was characterized according to its Tm.

JCPyV was detected using real-time qPCR with JE3 primers and a JE3 probe set (JE3-(Mad-1)F5′-ATGTTTGCCAGTGATGATGAAAA-3′, JE3-(Mad-1)R5′-GGAAAGTCTTTAGGGTCTTCTACCTTT-3′ and JE3-(Mad-1)Probe 5′-FAM-AGGATCCCAACACTCTACCCCACCTAAAAAGA-BHQ1-3′) according to a previously described protocol [23, 24]. The PCR reaction contained 1X Brilliant® II QRT-PCR Master Mix (Agilent Technologies Santa Clara, CA, USA), 0.5 μM of each primer, 0.15 μM of probe and 5 μL of DNA in a total volume of 25 μL. The amplification conditions were as follows: 95 °C for 10 min, followed by 50 cycles with denaturation at 95 °C for 15 s and annealing/extension at 60 °C for 1 min. The sensitivity for JCPyV and BKPyV detections by qPCR was 40 viral copies in 20 μL of reaction mix.

### Large T antigen (TAg) transcript detection

RNA purification was carried out using the High Pure RNA paraffin kit (Roche), following the manufacturers’ instructions. The RNA was suspended in 50 μL of TE (10 mM Tris-Cl, 1 mM EDTA) and stored at −80 °C until use. The RNA was incubated for 1 h at 37 °C with RQ1 DNAse and the enzyme was inactivated by incubation at 70 °C during 10 min. The cDNA preparation was made in accordance with the conditions established by the manufacturer using M-MLV reverse transcriptase (Promega). Negative controls without M-MLV were included. The reaction mixture was incubated at 37 °C for 1 h and stored at −20 °C. We used GAPDH amplification as a constitutive expression control, according to a previously reported protocol [25]. The amplification of TAg transcripts was carried out using the following primers: BKF: 5′-AGC CAC ACC CAG TTC AAA AG-3′and BKR: 5′-AAA CAA CAC TAG CTG CAG GG-3′. The 25-μL final volume reactions contained 1X Buffer (Promega), 0.4 μM of each primer, 1.5 μM MgCl_2_ and 1 μL of cDNA template. The amplification program was as follows: initial denaturation at 95 °C for 5 min followed by 45 cycles consisting of denaturation at 94 °C for 1 min, annealing at 55 °C for 1 min and extension at 72 °C for 1 min, followed by a final extension at 72 °C for 5 min. The amplification products (98 bp) were stained with MasterSafe (Promega), visualized under UV transillumination and photographed.

### Statistical analysis

Statistical analysis was performed using the Fisher’s exact test with the Stata 13.0 software. A *p*-value of less than 0.05 was considered statistically significant.

## Results

In this study we determined the presence of HPV, BKPyV and JCPyV in PCas in Chilean patients. Sixty-nine PCa samples obtained from patients at two public hospitals in Santiago, Chile were selected. Table 1 shows the clinical-pathological characteristics of patients included in this study. The mean age was 63 ± 6 years and the mean Gleason score of the tumors was 6.98 ± 1.10, with a range of 2 to 9. Age did not differ between the two hospitals (*p* = 0.336). However, we found that the Gleason score was significantly different between the two hospitals; patients treated at the J. J. Aguirre Hospital had a higher Gleason score compared to patients treated at the Barros Luco Hospital (*p* = 0.006).

**Table 1:** Clinical and pathological characteristics of study patients by hospital

		Number of subjects (%)		
		J. J. Aguirre	Barros Luco	*p*-value
Total		38 (100)	31 (100)	
Age	<65	21 (55.3)	13 (41.9)	0.336
	≥65	17 (44.7)	18 (58.1)	
Gleason	Low	7 (18.4)	17 (54.8)	0.006*
	Medium	20 (52.6)	8 (25.8)	
	High	11 (29.0)	6 (19.4)	

*Indicates a statistically significant difference by hospital

**Fig. 1:**
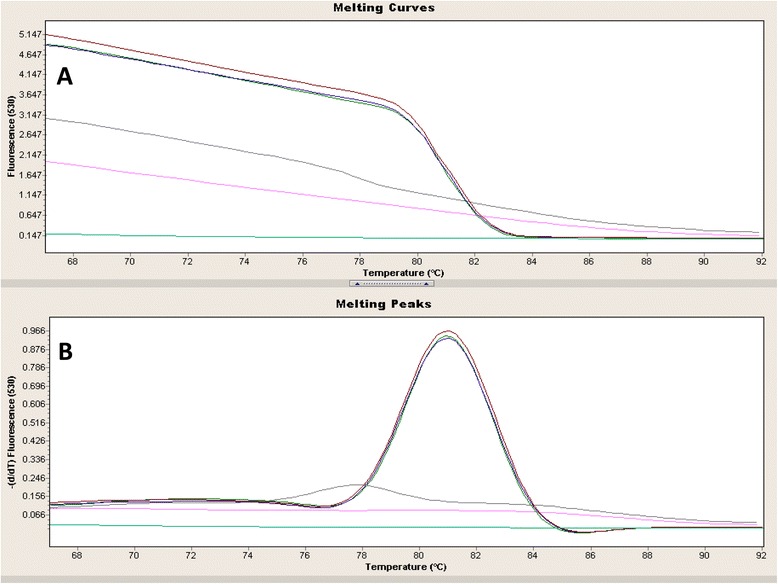
Dissociation curves and melting analysis of BKPyV positive and negative cases. The observed Tm was 80.8ºC.

Amplification of a 110 bp fragment of b-globin gave a similar yield in all samples. Using a highly sensitive genotyping assay (TS-MPG), HPV genomes were not detected in any of the samples. In addition, the absence of HPV genomes in all samples was confirmed by an independent GP5+/6+ PCR assay [26]. In contrast, for polyomaviruses we found that 6/69 (8.7 %) samples were positive by qPCR for BKPyV. Table 2 shows BKPyV detection and its association with clinical-pathological features. No statistically significant differences were found between age (*p* = 0.198), Gleason score (*p* = 0.268) or hospital (*p* = 0.402) and BKPyV presence in PCa. The dissociation curves for BKPyV and melting analysis of BKPyV positive and negative cases are shown in the Fig. 1.

**Table 2:** BKPyV presence in prostate cancer in relation to clinical-pathological features

		Number of subjects (%)		
		BKPyV (−)	BKPyV (+)	*p*-value
Total		63 (100)	6 (100)	
Age	<65	33 (52.4)	1 (16.7)	0.198
	≥65	30 (47.6)	5 (83.3)	
Gleason	Low	22 (34.9)	2 (33.3)	0.268
	Medium	27 (42.9)	1 (16.7)	
	High	14 (22.2)	3 (50.0)	
Hospital	Barros Luco	27 (42.9)	4 (66.7)	0.402
	José J. Aguirre	36 (57.1)	2 (33.3)

Finally, we determined the expression of the TAg at the RNA level in BKPyV positive cases. Table 3 shows the expression of TAg in BKPyV positive cases along with hospital, age and Gleason score.

**Table 3:** Large T antigen expression and clinical features for BKPyV positive prostate cancers

Patient	Hospital	Age	Gleason	TAg
1	BL	65	6	-
2	BL	65	7	-
3	JJA	67	8	+
4	JJA	57	8	-
5	BL	67	8	+
6	BL	68	6	-

*BL* Barros-Luco Hospital, *JJA* José Joaquín Aguirre Hospital

## Discussion

PCa is a biologically heterogeneous tumor affecting men that involves multiple factors in its etiology [27]. Several risk factors for PCa have been identified, such as age, familiar history, ethnicity, diet and environment [28]. In addition, molecular alterations involved in PCa development have been well characterized, and some alterations in specific pathways have been identified, such as the androgen receptor pathway, phosphatidyl inositol 3-kinase (PI3K) and functional loss of the prostate tumor suppressor NKX3.1, among others [29]. In this context, the potential role of HPV or HPyVs infections in the development of this malignancy has been investigated previously. It must be mentioned that in order to establish a causal relationship between HPV or HPyVs and PCa development, some questions need to be answered. First, is it biologically possible for HPV or HPyVs to be involved in prostate cancer development? In this respect, Choo et al. were able to immortalize prostate epithelial cells with HPV-16 E6/E7 oncogenes [30], and earlier experimental *in vitro* approaches demonstrated that HPV in collaboration with Ki-ras activation was able to transform prostate cells, raising the possibility of HPV-mediated oncogenicity [31]. It has also been suggested that BKPyV is a cofactor for PCa in its early stages, which is in agreement with the low occurrence of pRb and Rb1 mutations [12]. Moreover, BKPyV is oncogenic in hamster and mouse cells, where the continuous expression of a functional TAg is required [32]. Thus, it seems biologically possible that both viruses may be able to transform prostate cells and could potentially be involved in prostate carcinogenesis. The second question is: Is this biological relationship epidemiologically important to PCa development? This is a controversial topic for the reasons mentioned previously. In particular, the reported rate of HPV infection in PCa varies worldwide [9], and specifically it has been suggested that the male genital tract or the sperm may be a reservoir for the presence of this virus [33]. The role of HPV in PCa development has been evaluated in many studies [9]; however, findings have been inconclusive [34]. In the present study, using two different PCR-based approaches we were not able not detect HPV DNA sequences in PCa samples. Although our results suggest that HPV is probably not related to this malignancy, we cannot strictly rule out a “hit and run” mechanism, as has been previously suggested for the HPV-18 genotype [35]. JCPyV has previously been reported in benign prostate hyperplasia (BPH) and in PCa, suggesting that it is probably a bystander infection in the prostate [10]. Moreover, it was reported that JCPyV was frequently present in normal prostate specimens, suggesting that this virus replicates in the prostate epithelium [36]. In this study we were not able to detect JCPyV infection in any of the PCa specimens, suggesting that this virus is not related to the development of this disease. BKPyV was detected in 8.7 % of PCa specimens, and 33 % of BKPyV positive specimens expressed the TAg. It has been previously reported that this oncoprotein is able to bind and inactivate pRb and p53 tumor suppressor proteins, frequently downregulated in cancer, and specifically in viral-induced tumors [37]. Thus our results suggest that in the 33 % of BKPyV positive cases an oncogenic role of BKPyV is plausible. However, the limitations of this study are that our methodological approach does not allow us to determine if the viral infection is present in each tumor cell, as the original specimen contains adjacent non-tumor cells. The use of microdissection or *in situ* methodological approaches will be necessary in future studies.

Given our findings, a third question arises: Is the presence of BKPyV in PCa clinically relevant? In this respect, it is plausible that the presence of oncogenic viruses with functional activity is related to changes in the behavior of the tumor, and consequently be related to clinical observations. It has been previously established that patients positive for high-risk HPV with oropharyngeal carcinomas have better outcomes and higher rates of survival compared to HPV negative patients [38]. However, such a relationship for viral infections in the prostate, and specifically for BKPyV, has not been established. In our study we did not find a statistically significant association between BKPyV infection and age, even though 87 % of BKPyV infections were detected in subjects over 65 years of age. This finding is compatible with the reactivation of this virus in the elderly or those under immunosuppressed conditions. In addition, 50 % of BKPyV positive patients showed a high Gleason score as compared to 22 % of BKPyV negative patients, although this difference was not statistically significant. Although this virus is acquired in early childhood, the transmission route is unknown, but is likely through oral or gastrointestinal mucosa, and disseminated into the kidneys where the virus develops a persistent infection [39, 40]. In this respect it has been suggested that rearranged noncoding control regions (NCCRs) of polyomaviruses altering the number and composition of transcription factor binding sites (TFBSs) are involved in silent persistence and reactivation [41]. In addition, it has been reported that BKPyV downregulates Toll like receptor 9 (TLR9) raising the possibility that such downregulation is involved in viral persistence [42]. In fact, it was established that 90 % of the population is serologically positive for BKPyV infection [43].

In conclusion, in this study we determined that BKPyV was present in a discrete number of prostate cancers in Chilean patients, with partial detection of TAg antigen in BKPyV positive specimens, suggesting a causal association in a subset of cases. More studies are warranted to elucidate the clinical significance of these findings.
